# 

**Published:** 1891-10-24

**Authors:** 


PRICE ONE PENNY.

No. 265, Vol. XI.—October 24th, 1891.

[Registered at the General Post Office as a Newspaper for transmission in the United Kingdom and Abroad.]

WITH EXTRA NURSING SUPPLEMENT

Per Annum, 6s. 6d.; post free

Monthly Parts, 6d.; post free 8d.

Ditto per Annum, 8s., post free.

### THE HOSPITAL

GLASGOW WESTERN INFIRMARY — ST THOMAS'S HOSPITAL — NORFOLK AND NORWICH HOSPITAL

A WEEKLY JOURNAL OF
Science, Medicine, Nursing and Philanthropy.

SATURDAY, OCTOBER 24th, 1891.

**Medical Politics** - 37
**Passing Topics.**—Cheap Benevolence - 38
**The Doctor's Armchair.**—Theosophy and Mrs. Besant - 39
**Some Scientific Aspects of Insanity.** I.—Brain Tissue - 40
**Everybody's Page** - 41
**Notes and News** - 42
**General Charities** - 43
**Scraps and Gleanings** - 43
**Annotations.**—The Practical Man—A Pillar of Fortune—"I go on for Ever" - 44
**The Practitioner's Mirror and Retrospect—**
MEDICINE.—New Methods of Treatment in Phthisis. IV.—Transfusion of the Blood of Refractory Animals, 45; Obesity: Its Causes and Treatment - 46
OPHTHALMOLOGY.—Ophthalmia Neonatorum - 47
PRACTITIONERS' BOOKSHELF.—Atlas of Clinical Medicine - 47
NEW DRUGS, APPLIANCES, AND THINGS MEDICAL.—The Baby Carrier - 47
**The Hospital Annual** - 48
**The Student of Medicine.**—Appointments—Vacancies

EXTRA SUPPLEMENT—THE NURSING MIRROR.

**En Passant.**—Nurses in Belvedere—Nurses for the Middle Classes—Nurses' Instruments—Short Items—Improvements at Norwich—Attendants in Trouble - xix
**Lectures on Surgical Ward Work and Nursing.**—By ALEX. MILES, M.D. Edin. Lecture XXXVIII.—Some Instruments Used in Connection with Operations on Bone - xx
**Appointments** - xx
**Help for the Dying** - xxi
**A Bed for a Sick Nurse** - xxi
**For Reading to the Sick.**—Trifles - xxi
**A Nurse in Natal** - xxii
**Everybody's Opinion.**—Six Months' Certificates—Nurses as Patients—James Payn's Nurses—All Saints' Nurses - xxiii
**Presentations** - xxiii
**Wants and Workers** - xxiii
**Off Duty.**—From Pharmacy to Farming—(*continued*) - xxiv
**Notes and Queries** - xxiv
**Amusements and Relaxation** - xxiv

